# Tailoring the Lamellarity of Liposomes Prepared by Dual Centrifugation

**DOI:** 10.3390/pharmaceutics15020706

**Published:** 2023-02-20

**Authors:** Jonas K. Koehler, Lars Gedda, Leonie Wurster, Johannes Schnur, Katarina Edwards, Heiko Heerklotz, Ulrich Massing

**Affiliations:** 1 Institute of Pharmaceutical Sciences, University of Freiburg, 79104 Freiburg im Breisgau, Germany; 2 Department of Chemistry-Ångström, Uppsala University, 75237 Uppsala, Sweden; 3 Leslie Dan Faculty of Pharmacy, University of Toronto, Toronto, ON M5S 3M2, Canada; 4 Signaling Research Centers BIOSS and CIBBS, University of Freiburg, 79104 Freiburg im Breisgau, Germany; 5 Andreas Hettich GmbH & Co. KG, 78532 Tuttlingen, Germany

**Keywords:** dual centrifugation, liposomes, lipodisks, Cryo-EM, inaccessible surface, lamellarity, multilamellar vesicles, small multilamellar vesicles, drug delivery

## Abstract

Dual centrifugation (DC) is a new and versatile technique for the preparation of liposomes by in-vial homogenization of lipid-water mixtures. Size, size distribution, and entrapping efficiencies are strongly dependent on the lipid concentration during DC-homogenization. In this study, we investigated the detailed structure of DC-made liposomes. To do so, an assay to determine the ratio of inner to total membrane surfaces of liposomes (inaccessible surface) was developed based on either time-resolved or steady-state fluorescence spectroscopy. In addition, cryogenic electron microscopy (cryo-EM) was used to confirm the lamellarity results and learn more about liposome morphology. One striking result leads to the possibility of producing a novel type of liposome—small multilamellar vesicles (SMVs) with low PDI, sizes of the order of 100 nm, and almost completely filled with bilayers. A second particularly important finding is that VPGs can be prepared to contain open bilayer structures that will close spontaneously when, after storage, more aqueous phase is added and liposomes are formed. Through this process, a drug can effectively be entrapped immediately before application. In addition, dual centrifugation at lower lipid concentrations is found to produce predominantly unilamellar vesicles.

## 1. Introduction

Vesicular phospholipid gels (VPGs) are highly concentrated liposomal formulations that can be prepared from concentrated aqueous phospholipid mixtures by high-pressure homogenization (HPH) or dual centrifugation (DC). VPGs can be used as versatile and well-tolerated depot formulations, as reviewed by Tian et al. [1]. On the other hand, VPGs are also the starting material for the preparation of conventional liposomal formulations by simple dilution. A unique property of liposomes prepared from VPGs is their unusually high encapsulation efficiency (EE) of water-soluble compounds, up to roughly 70%, which can be explained by the high ratio of liposomal-entrapped aqueous phase to non-entrapped aqueous phase within a VPG [2].

Dual centrifugation (DC) is a new technique to rapidly prepare VPGs which can be converted to conventional liposomal dispersions by dilution as needed [3,4]. Dual centrifugation has recently been used for an increasing number of pharmaceutical applications. Deuringer et al. used DC for the preparation of reconstituted high-density lipoprotein nanoparticles loaded with an immunosuppressant drug, everolimus [5]. Langer et al. prepared albumin-stabilized mitotane nanoparticles by DC for the treatment of adrenocortical carcinoma [6]. DC was also used recently for the nanomilling of poorly soluble drugs such as fenofibrate [7] and itraconazole [8,9]. Steiner et al. used DC to prepare solid lipid nanoparticles [10] and were able to embed the lipid nanoparticles in orodispersible films [11]. DC was also used for the preparation of solid liposomal formulations (matrix liposomes) [12,13] and for the small-scale preparation of perfluorocarbon-nanoemulsions [14]. However, the most important use of DC currently is for the preparation of liposomes.

The DC-homogenization of concentrated phospholipid dispersions to obtain liposomes is usually performed in small and disposable vials, such as 2 mL polypropylene twist-top vials, and supported by the addition of ceramic beads. For DC, the oblong sample vials are positioned horizontally in the dual centrifuge instead of the usual vertical positioning. During DC, the sample vial is centrifuged at high speed and simultaneously turned around a second rotation axis. This additional rotation in combination with its horizontal positioning causes a rapid change in the direction of the high centrifugal acceleration inside the turning sample vial. The resulting powerful movement of the viscous phospholipid dispersions, including the beads moving from the top to bottom of the vial and vice versa, results in strong shear forces and consequently the homogenization of the dispersions. Thus, DC is also used for nanomilling or tissue disruption [4,7].

Dual centrifugation has many advantages in the production of VPGs and the corresponding liposomes. The process is carried out in closed disposable vials (“in-vial homogenization”), making it very simple and safe, since contact with the product is avoided and cleaning of the device is not necessary. The use of sterile vials allows for the easy preparation of sterile VPGs/liposomes. At the same time, DC-homogenization is gentler compared to high-pressure homogenization, since the energy input is lower than in HPH, but the number of homogenization events is much higher [3]. In addition, a dual centrifuge can be cooled.

Since the parallel processing of up to 40 different formulations in very small batches is possible, DC is an attractive tool for screening for improved lipid compositions and manufacturing conditions. In a previous work [2], the DC-based liposome screening process was optimized by using a simplified procedure to determine the EE-values, allowing for the simultaneous and rapid determination of entrapped and non-entrapped calcein using time-resolved fluorescence spectroscopy, omitting the time-consuming separation of the unentrapped marker. The new EE determination could be performed with the same sample used for measuring liposome size and size distribution (PDI), which further speeds up the screening process. Finally, it was shown by differential scanning calorimetry (DSC) that the preparation of a molecularly dispersed lipid mixture (the so-called “lipid film”) prior to liposome preparation is no longer necessary and that the lipids can simply be used as a macroscopic mixture, which may also contain cholesterol. The screening results can be arranged in a “liposome screening diagram” clearly showing at which phospholipid-concentrations the optimal size-, PDI-, or EE-values can be achieved [2].

A liposome screening diagram can be divided into three typical sections regarding the lipid concentrations used for liposome preparation: In the first section, up to about 20% to 30% lipid concentration, the liposome sizes decrease, and the EE-values increase with the increasing lipid concentration. In the following section, from about 20% to 30% up to 60% to 70% lipid concentration, the liposome sizes are at their minima and fairly constant, and only the PDI-values decrease. The EE-values in this section are high and also quite constant. In the third section, starting at lipid concentrations of >60% to 70%, the EE-values drop, and the liposome sizes and PDI-values increase [2].

Since with increasing lipid concentrations of >20% to 30%, the lipid content is higher than necessary to completely fill the volume of the resulting VPG with unilamellar vesicles, we expect the formation of multilamellar vesicles. Furthermore, at even higher lipid concentrations of >60% to 70%, the amount of water becomes too low to fully hydrate the phospholipid head groups and fill the spaces between and within liposomes [2]. It is reasonable then to assume that the limitation in available water could result in VPGs consisting of lipid structures that are no longer vesicular. In the present study, these relationships were investigated in more detail. Hence, the vesicle morphology of DC-made VPGs, as well as the morphology of the resulting liposomes, were studied in relation to the lipid concentration used for DC-homogenization. For this, a fluorescence-based assay was established based on dithionite quenching [15,16] to assess liposome lamellarity by measuring the relative amount of inaccessible surface (IAS) of the (multilamellar) vesicles. The results of the assay were confirmed using cryo-EM, which provided more detailed information concerning the lamellarity and overall structure of the liposomes and thus the correlation between IAS and lamellarity. To the best of our knowledge, there is no publication available showing cryo-EM of liposomes as dependent on the lipid concentration used during DC-homogenization.

## 2. Materials and Methods

### 2.1. Materials

Hydrogenated egg phosphatidylcholine (HEPC; ≥98%; trade name Lipoid: EPC-3), 1,2-dipalmitoyl-sn-glycero-3-phosphocholine (DPPC; ≥99%), 1,2-distearoyl-sn-glycero-3-phosphocholine (DSPC; ≥99%), and N-(Carbonyl-methoxypolyethylene glycol-2000)-1,2-distearoyl-sn-glycero-3-phosphoethanolamine, sodium salt (DSPE-PEG_2000_ (PEG); ≥98%) were kindly provided by Lipoid GmbH (Ludwigshafen, Germany). 1,2-distearoyl-sn-glycero-3-phosphoethanolamine-N-(7-nitro-2-1,3-benzoxadiazol-4-yl, ammonium salt) 18:0 NBD-DSPE (≥99%, product number: 810141P) was obtained from Avanti Polar Lipids (Birmingham, AL, USA).

Calcein (product number: C0875) and cholesterol (≥99%) were obtained from Sigma-Aldrich (St. Louis, MO, USA). Sodium dithionite (CAS-No: 7775-14-6, EMSURE^®^, for analysis) was obtained from Merck (Merk KGaA, Darmstadt, Germany). TRIS (hydroxymethyl)aminomethane (TRIS; ≥99.9%) and ethylenediaminetetraacetic acid (EDTA; ≥99%) were obtained from Carl Roth GmbH (Karlsruhe, Germany).

### 2.2. Methods

#### 2.2.1. Preparation of Liposomes with Prior Lipid Film Preparation

Molecularly dispersed lipid mixtures (lipid films) were prepared by dissolving the lipids in chloroform to produce a solution of 200 mM total lipid. Aliquots were pipetted into 2 mL conical screw cap vials (Sarstedt AG & Co. KG, Nümbrecht, Germany, Type 72.693.005), and the solvent was removed in a vacuum centrifuge (RVC 2–18, Martin Christ Gefriertrockungsanlagen GmbH, Osterode, Germany) equipped with a vacuum pump (MZ 2C, Vacuubrand GmbH + Co KG, Wertheim, Germany) at 45 °C for 2–4 h. To ensure the complete removal of the solvent, the lipid films were transferred to a vacuum desiccator equipped with a high-vacuum pump (RD 4, Vacuubrand GmbH + Co KG, Wertheim, Germany) for at least 12 h.

Liposome samples were prepared as previously described [2]. In brief, 600 mg zirconium oxide beads (1.5 mm) (SiLibeads^®^ Type ZY-P Pharma, Sigmund Lindner GmbH, Warmensteinach, Germany) and the necessary amount of aqueous phase were added to the vials already containing the lipids. For samples with NBD-DSPE, a TRIS buffer with pH 7.4 (10 mM TRIS, 0.5 mM EDTA) was used. TRIS buffer (10 mM TRIS, 0.5 mM EDTA, pH 7.4) was used for the cholesterol-containing samples. For the cholesterol-free samples, 25 mM NaCl was added to the TRIS buffer for osmotic balancing of the 10 mM sodium dithionite. For the samples with calcein, a TRIS buffer (10 mM TRIS, 0.5 mM EDTA) with a pH of 8.5 was used. The desired lipid concentration (10–80%) was achieved by mixing the 10–80 mg lipid mixture with the corresponding 90–20 µL of aqueous buffer, respectively. The lipid concentration (%) is given as w/v in the VPG. For convenience, we approximated that 1 mg lipid per 100 µL dispersion represents 1% lipid concentration, due to a density close to 1 g/mL for both the buffer and phospholipids. Liposome preparation was performed using a dual centrifuge (DC; ZentriMix 380 R (Andreas Hettich GmbH & Co. KG, Tuttlingen, Germany)) equipped with adapters for processing 40 vials in parallel (Standard DC-settings: 2350 rpm, 30 min, 20 °C for cholesterol-containing lipid mixtures and 2350 rpm, 30 min, 40 °C for the cholesterol-free lipid mixtures). The adjustment of the temperature of the dual centrifuge concerns the rotor and the surrounding air. Due to the strong homogenization forces, the sample typically heats up by approx. 20 °C compared to the temperature set on the instrument. The resulting VPGs were typically diluted to a liposomal dispersion with TRIS buffer pH 7.4 (10 mM TRIS and 0.5 mM EDTA) for cholesterol-containing samples or an additional 25 mM NaCl for cholesterol-free samples, in the ratio 3:1 (*v/v*) by DC (1500 rpm, 20 °C) for 2 min. For all lipid mixtures, a batch size of 100 mg vesicular phospholipid gel (VPG) was prepared to standardize the influence of the beads and wetting of the vial surface.

#### 2.2.2. Liposome Preparation without Lipid Film Preparation

The lipid components (dry powder form) were individually weighted in the vial. The beads and buffer were added according to Section 2.2.1 and prepared by DC at 2350 rpm, for 30 min, and at 20 °C.

#### 2.2.3. Liposome Size and Size Distribution

Dynamic light scattering (DLS, Zetasizer Nano-ZS, Malvern Instruments, Worcestershire, UK) was used to determine the intensity-weighted hydrodynamic diameter (Z-average) and polydispersity index (PDI) of the DC-produced liposomes. Liposomal dispersions were diluted with TRIS buffer (viscosity: 0.8749 mPa∙s; refractive index: 1.330). The attenuator was set automatically to reach a count rate of about 150–250 kcps. The instrument evaluated the backscattering intensity from a 4 mL polystyrene cuvette with a layer thickness of 10 mm (Sarstedt AG & Co. KG, Nümbrecht, Germany) at an angle of 173°. Three measurements with an automatically selected number of scans (10–17 scans, 10 s per scan) were taken for each sample.

#### 2.2.4. Inaccessible Surface Measurements

VPGs containing a nitrobenzoxadiazole (NBD)-labeled lipid were diluted to a lipid concentration of 0.05 mM with TRIS buffer pH 7.4 using DC (1500 rpm, 20 °C, and 2 min). Samples with quencher contained 10 mM sodium dithionite. Samples were prepared from a 1 M sodium dithionite stock containing 1 M TRIS; the sodium dithionite stock was prepared fresh on the same day as the quenching experiments due to the instability of sodium dithionite in an aqueous solution. The liposomal dispersion was added to the sodium dithionite-containing buffer 10 min before measurement and all measurements were conducted in an anti-photobleaching setup, whenever possible. Three different measurement methods, described below, were used to determine the loss in fluorescence intensity caused by the reduction of the NBD-headgroup.

##### Time-Resolved Fluorescence (TRF) Measurements: FT100

**Figure 1:**
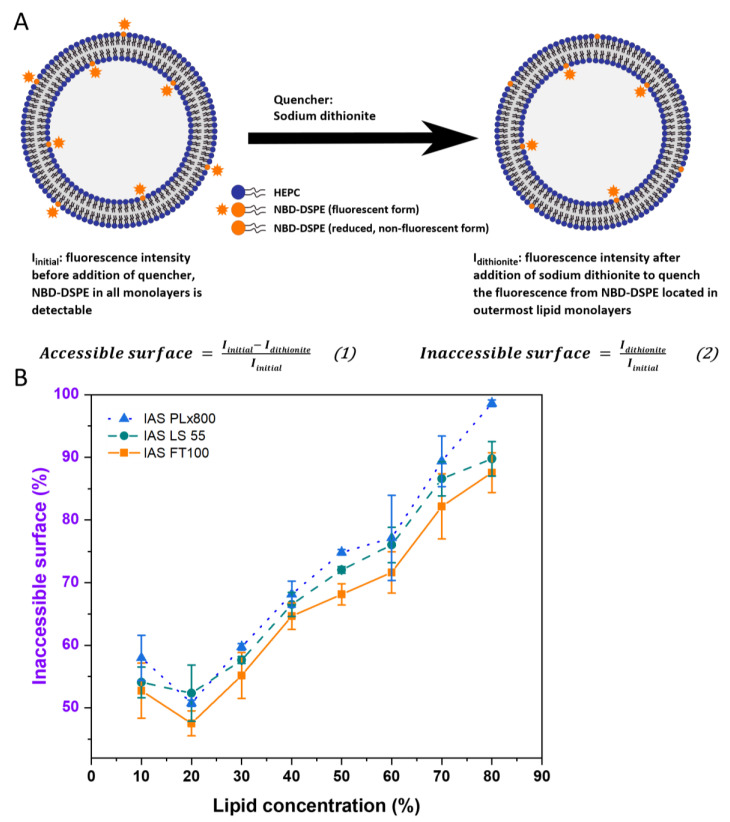
(**A**): Schematic illustration of the inaccessible surface (IAS) assay (for details, see text). (**B**): Inaccessible surface (IAS) assay with different fluorescence determination methods (averaged time-resolved fluorescence: FT100, steady-state fluorescence in a cuvette: LS55 and steady-state fluorescence in a 96-well plate: PLx800) using the example of HEPC/Chol/NBD-DSPE 54.9/45/0.1 (mol%) liposomes prepared by DC (2350 rpm, 30 min, and 20 °C) and redispersed by DC (1500 rpm, 2 min, and 20 °C). Data are shown as the mean ± SD (n = 3).

Time-resolved fluorescence (TRF) measurements were performed using the fluorescence lifetime spectrometer FluoTime 100 (PicoQuant, Berlin, Germany). The excitation source was a modular laser diode (LDH-P-C-470) with a wavelength of 470 nm (±10 nm), pulsed at a repetition rate of 5 MHz. The laser was operated with the laser driver PDL 800-D (PicoQuant, Berlin, Germany). Decay curves were recorded at a 515 nm wavelength by using time-correlated single-photon counting (TCSPC). For each sample, the measurement conditions were set to a peak count of at least 10,000 photons for a sample without a quencher. The measuring period was set to 120 s and the measurement conditions were kept the same for the samples with and without the added quencher. Fluorescence decay curves were fitted by FluoFit software (PicoQuant, Berlin, Germany) by reconvolution of a biexponential fitting function with the instrument response curve recorded using a Ludox scattering standard. IAS was calculated using total counts according to Formula (2) (Figure 1), the total counts were calculated by the determined fit parameters.

(1) $$Accessible\;surface=\frac{I_{initial}-I_{dithionite}}{I_{initial}}$$

(2) $$Inaccessible\;surface=\frac{I_{dithionite}}{I_{initial}}$$

##### Steady State Fluorescence Measurements: LS55

Steady-state fluorescence measurements were conducted using a PerkinElmer LS55 fluorescence spectrometer (PerkinElmer Inc., Waltham, MA, USA). A spectral bandpass of 460 nm (±10 nm slit) and 535 nm (±10 nm slit) were used for excitation and emission, respectively. The fluorescence intensity at 25 °C was measured for samples with and without sodium dithionite.

##### Steady State Fluorescence Measurements in a Plate Reader: PLx800

Steady-state fluorescence measurements in a black 96-well microtiter plate were conducted using the BioTek PLx800 fluorescence reader (BioTek Instruments, Inc., Winooski, VT, USA). The measurements were each performed in triplicate with a total volume of 200 µL per vial. A 485 nm excitation filter and a 528 nm emission filter were used. 

#### 2.2.5. Uptake of Calcein into Open Lamellar Structures

Calcein-free VPGs were prepared by DC-homogenization (2350 rpm, 30 min, and 20 °C) of the respective lipid films and the respective volume of TRIS buffer (10 mM TRIS, 0.5 mM EDTA, pH 8.5). In the next step, 60 mM calcein buffer was added to the VPG and homogeneously dispersed by DC for HEPC/Chol 55/45 up to a total lipid concentration of 40% with 1000 rpm, for 2 min, and at 20 °C; for HEPC/Chol/PEG 50/45/5 up to 20% for VPGs with a lipid concentration lower than 55%; and up to 40% for VPGs with a lipid concentration of 55% and higher with 600 rpm, for 2 min, and at 20 °C. In the third step, the calcein/VPG mixture was diluted with TRIS buffer by DC (500 rpm, 2 min, 20 °C).

After preparation, the calcein-containing liposome dispersions were diluted in the ratio of 1:5000 prior to the *EE* determination as previously described [2]. Time-resolved fluorescence (TRF) measurements were conducted with the fluorescence lifetime spectrometer FluoTime 100 (PicoQuant, Berlin, Germany). The excitation source was a modular laser diode (LDH-P-C-470) with a wavelength of 470 nm (±10 nm), pulsed at a repetition rate of 20 MHz. The laser was operated with the laser driver PDL 800-D (PicoQuant, Berlin, Germany). Decay curves were recorded at a 515 nm wavelength by using time-correlated single-photon counting (TCSPC). For each sample, the measurement conditions were set to a peak count of at least 10,000 photons for each sample by varying the transmission (0.1–1%). The measuring period was set to 30 s. Fluorescence decay curves were fitted with FluoFit software (PicoQuant, Berlin, Germany) by a biexponential function. Encapsulation efficiency (EE) was calculated according to the method described previously [2]. In brief, with the TRF method, it is possible to determine simultaneously the amount of entrapped and free calcein, and thus the time-consuming and error-prone separation of free calcein is not necessary.

#### 2.2.6. Cryo-EM Specimen Preparation and Analysis

The samples for the cryo-EM analysis were prepared by DC as described in Section 2.2.1, but without the headgroup fluorescence label NBD-DSPE and with plain TRIS buffer. Samples were analyzed using cryo-EM as described earlier by Almgren et al. [17]. Briefly, samples were equilibrated at 25 °C and a high relative humidity in a climate chamber. A total of 1 µL of each sample was deposited on a carbon-sputtered copper grid covered with a perforated polymer film. Then, excess liquid was removed by blotting with filter paper, leaving a thin film of the solution on the grid. The specimen was immediately vitrified in liquid ethane and transferred to the microscope, continuously kept below −160 °C, and protected against atmospheric conditions. Analyses were performed with a Zeiss Libra 120 transmission electron microscope (Carl Zeiss AG, Oberkochen, Germany) operating at 80 kV in zero-loss bright-field mode. Digital images were recorded under low-dose conditions with a BioVision Pro-SM Slow Scan CCD camera (Proscan Elektronische Systeme GmbH, Scheuring, Germany).

## 3. Results

### 3.1. Morphology of Cholesterol-Containing Liposomes

Previous investigations showed that important characteristics of liposomes, such as entrapping efficiencies for water-soluble compounds, size, and size heterogeneity, can be modulated through a conscious choice of the lipid concentration used during the DC-homogenization [2]. To further explore the influence of lipid concentration on liposome morphology, we developed an assay to evaluate liposome lamellarity by measuring the ratio of the inner to outer membrane surface areas (inaccessible surface, IAS) of the vesicles. Selected samples were analyzed by cryo-EM to directly visualize and learn more about the size distribution and detailed structure of the liposomes.

#### 3.1.1. Fluorescence-Based Inaccessible Surface Assay

To determine the inaccessible surface (IAS) of DC-prepared liposomes, 0.1 mol% of the fluorescence-labeled lipid NBD-DSPE was homogeneously dispersed in the lipid mixture used for liposome preparation. The fluorescence signal of NBD-DSPE at the outer (accessible) surface of the liposomes was quenched by adding the essentially membrane-impermeable sodium dithionite, which reduces the nitro-group of NBD to an amino-group (Figure 1A) as described earlier by McIntyre and Sleight [15]. Determination of the NBD-fluorescence intensity with and without the addition of sodium dithionite allowed for the calculation of the inaccessible surface (IAS) (Formula (2)) and the accessible surface (AS) (Formula (1)) of the liposomes [16].

With increasing IAS, the liposomes consisted of a higher number of lamellae, i.e., the lamellarity increases. Calculating the exact number of lamellae was not possible due to several reasons, such as non-homogeneous size distribution, the occurrence of intravesicular vesicles, and a decrease in the diameter with each additional lamella.

The fluorescence intensities used for IAS measurements were determined with three different types of fluorescence spectrometers and compared with each other. The three instruments (averaged time-resolved fluorescence measurement (FT100), steady-state fluorescence spectrometer using cuvettes (LS55), and MTP-fluorescence photometer (PLx800)) resulted in slightly different absolute results, but the trend for AS and IAS were the same and thus sufficient to characterize the lamellarity of liposomes (Figure 1B).

**Figure 2:**
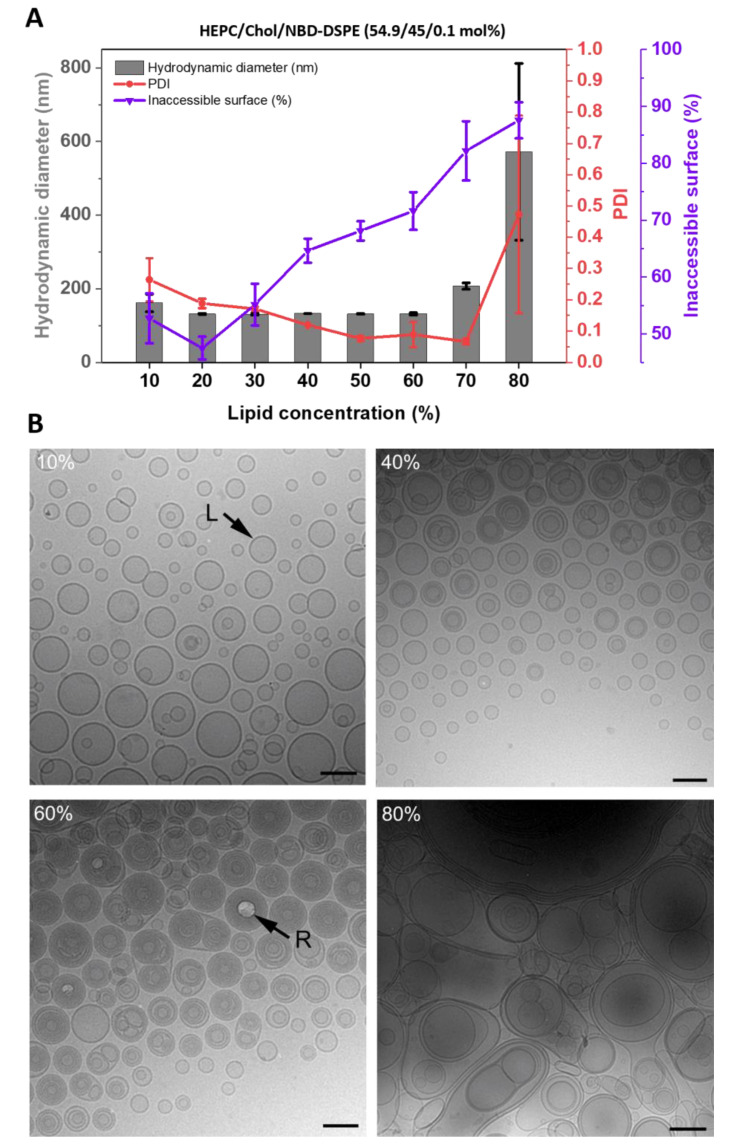
(**A**): Inaccessible surface, hydrodynamic diameter, and PDI depending on the lipid concentration used for the DC-homogenization of HEPC/Chol/NBD-DSPE 54.9/45/0.1) (mol%) lipid/buffer mixture; the resulting liposomes were measured by a fluorescence-based IAS-assay and DLS. Liposomes were prepared by DC at 2350 rpm, for 30 min, and at 20 °C, and redispersed by DC at 1500 rpm, for 2 min, and at 20 °C with TRIS buffer. Data are shown as the mean ± SD (n = 3). (**B**): Cryo-EM images of the liposome formulations shown in the IAS-assay figure above but without the headgroup-labeled fluorescence marker NBD-DSPE. Cryo-EM images of HEPC/Chol 55/45 (mol%) consisting of liposomes prepared by DC at 10%, 40%, 60%, and 80% lipid concentrations (VPG). Arrows denoted (L) indicate liposomes and those labeled (R) indicate radiation damage due to multiple layers containing liposomes. Scale bar = 100 nm.

Figure 2A shows the inaccessible surface (IAS), hydrodynamic diameter, and PDI of HEPC/Chol/NBD-DSPE 54.9/45/0.1 liposomes as a function of the lipid concentration used to prepare the VPGs/liposomes by DC-homogenization. The trend for the IAS in relation to the lipid concentration could be divided into three parts, as already seen for the trend of EE-values [2]. With the increasing lipid concentration of the lipid/buffer mixture, the IAS remains rather constant from 10% to 30% lipid concentration and is continuously increasing from 30% to 80% lipid concentration (Figure 2A). As shown in [2], the EE-*value* increases from 10% to 30% lipid concentration, reaches a plateau area from 30% to about 50%, and decreases from 50% to 80% lipid concentration. The liposomes tend to be larger and more polydisperse in size when prepared from lipid/buffer mixtures with <30% or >70% lipid concentration (Figure 2A). These observations are compatible with a scenario in which the liposomes become increasingly multilamellar with increasing lipid concentration until, at some point, a major change in structure occurs.

#### 3.1.2. Cryo-EM

To confirm that the lamellarity of the liposomes depends on the lipid concentrations used for DC-homogenization, a selection of samples produced by DC-homogenization at different lipid concentrations was analyzed by cryo-EM (Figure 2B). Micrographs obtained from samples produced with 10% lipid displayed mainly unilamellar liposomes that were heterogeneous in size, with diameters ranging from about 150 nm down to <20 nm. A small population of liposomes that seemingly encapsulated one or two smaller liposomes was also observed, as well as some apparently hemifused liposomes. Samples produced with 40% lipid also contained a significant proportion of unilamellar liposomes, albeit their minimum size was larger (~40 nm) than what was observed for the 10% sample. Micrographs obtained from the 40% of samples also revealed a considerable population of multilamellar and multivesicular structures. Increasing the lipid concentration to 60% resulted in samples dominated by multilamellar liposomes of fairly homogeneous size (~100 nm) in which the lamellae were extremely densely packed. Finally, samples produced with 80% lipid displayed structures that were remarkably heterogeneous in both size and structure. More specifically, very large multilamellar and multivesicular structures, with sizes, in some cases, exceeding 1 μm observed to coexist with a few smaller, often bilamellar liposomes.

#### 3.1.3. Effects of Adding 5 mol% DSPE-PEG_2000_ to the HEPC/Chol Lipid Mixtures

**Figure 3:**
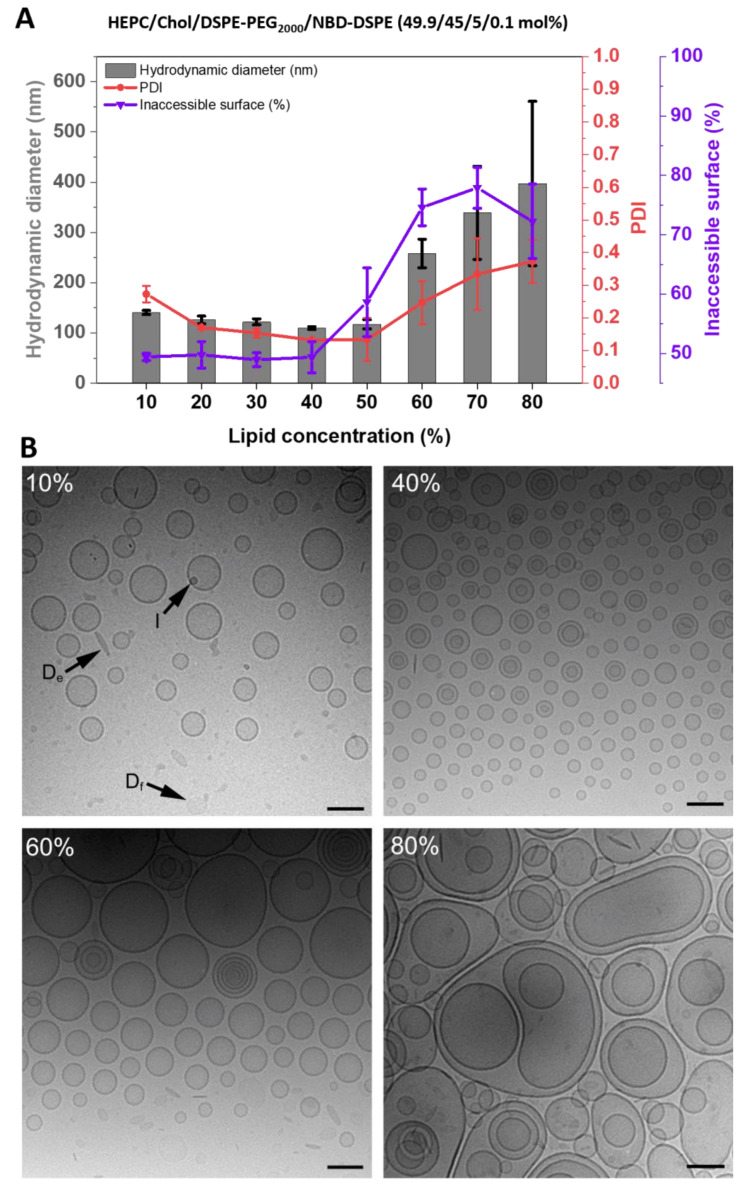
(**A**): Inaccessible surface, hydrodynamic diameter, and PDI depending on the lipid concentration used for the DC-homogenization of HEPC/Chol/DSPE-PEG_2000_/NBD-DSPE 49.9/45/5/0.1) (mol%) lipid/buffer mixture. The resulting liposomes were measured by fluorescence-based IAS assay and DLS. Liposomes were prepared by DC at 2350 rpm, for 30 min, and at 20 °C, and redispersed by DC at 1500 rpm, for 2 min, and at 20 °C with TRIS buffer. Data are shown as the mean ± SD (n = 3). (**B**): Cryo-EM images of HEPC/Chol/DSPE-PEG_2000_ 50/45/5 (mol%) liposomes prepared by DC at 10%, 40%, 60%, and 80% lipid concentrations (VPG). Arrows denoted (D_e_) indicate lipodisks viewed edge-on and lipodisks viewed face-on (D_f_); (I) indicates ice crystals. Scale bar = 100 nm.

The inclusion of 5 mol% DSPE-PEG_2000_ (PEG) into the membranes of HEPC/Chol-liposomes led to clear differences in the trend for the IAS with increasing lipid concentration (Figure 3). With increasing lipid concentration upon DC-homogenization, the IAS remained rather constant for 10% to 40% lipid concentration and continuously increased from 40% to 70% lipid concentration (Figure 3A). As shown in [2], the encapsulation efficiency (EE) increased from 10% to 20% lipid concentration, reached a plateau area from 20% to about 50%, and decreased from 50% to 80% lipid concentration. The liposome size tended to be larger and the liposomes were more polydisperse in size below 20% and above 50% lipid concentration (Figure 3A). Particularly noteworthy was the lower IAS in the range of 10% to 50% lipid concentration for the PEG-containing liposomes, as compared to the non-PEGylated liposomes (Figure 2A). Furthermore, the IAS decreased from 70% to 80% lipid concentration while the size further increased.

In addition, cryo-EM analyses revealed clear differences between the samples prepared in the absence and presence of DSPE-PEG_2000_ (Figure 2B and Figure 3B, respectively). First, the extremely small liposomes observed for the 10% lipid samples without PEG-lipid were not detected in the corresponding samples supplemented with the PEG-lipid. Second, all samples prepared in the presence of PEG-lipid displayed, irrespectively of lipid concentration, a population of circular bilayer fragments commonly referred to as PEG-stabilized lipodisks [18]. In the micrographs, the lipodisks can be observed face-on, edge-on, as well as at other orientations with respect to the incoming electron beam. Third, although increasing the lipid concentrations up to 40% or 60% tended to cause a gradual shift from unilamellar to multilamellar liposomes, the trend was less pronounced in the case of the PEG-supplemented samples, which supported the findings of the IAS-assay (see above). The population of multilamellar liposomes was clearly smaller for the PEG-containing samples, and the liposomes tended to contain fewer and less densely packed lamellae. Finally, for samples prepared using 60% and 80% lipid concentrations, the inclusion of the PEG-lipid had a noticeable effect on the liposome size distribution. Micrographs obtained from the 60% samples disclosed liposomes with a broader size distribution (50 to 300 nm) and an, on average, larger size when PEG-lipid was included. In the case of the 80% samples, the maximum size of the liposomes decreased from >1 μm without PEG-lipid down to about 500 nm upon inclusion of PEG-lipid.

#### 3.1.4. Open Membrane Stack Structures in VPGs

The measurements of the IAS, as well as the analyses based on DLS and cryo-TEM described in the previous sections, were all conducted on liposome dispersions, i.e., samples prepared by diluting the VPGs with buffer. In the case of VPGs prepared using high lipid concentrations, it is conceivable that structural changes may occur due to this dilution step. It is, for instance, plausible that the large liposomes observed in samples prepared with 80% lipid (Figure 2B and Figure 3B) form during the dilution step as a result of the hydration and closure of open membrane stacks present in the original VPGs. To support or refute this hypothesis, VPGs made without the marker molecule calcein were diluted with a calcein-containing buffer, which gives the potentially existing open lipid structures the chance to close and, at the same time, trap the dye. Already closed structures should not take up the dye.

**Figure 4:**
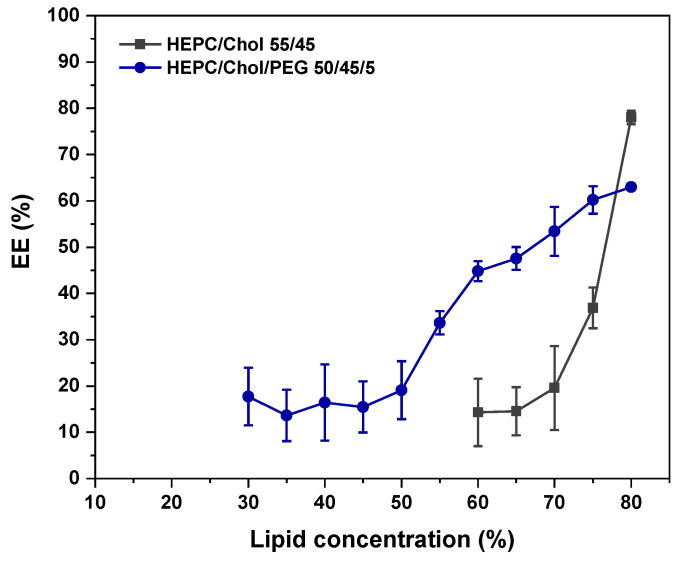
Uptake of calcein into calcein-free VPGs. The VPGs were prepared calcein-free with TRIS buffer (10 mM TRIS, 0.5 mM EDTA, pH 8.5) containing 100 mM NaCl by homogenization of the lipid film with the aqueous buffer by DC (2350 rpm, 30 min, and 20 °C). In the next step, 60 mM calcein buffer was added to the VPGs and homogeneously dispersed by DC. For HEPC/Chol 55/45 up to a total lipid concentration of 40% with 1000 rpm, for 2 min, and at 20 °C. For HEPC/Chol/PEG 50/45/5 up to 20% for VPGs with a lipid concentration lower than 55%, and up to 40% for VPGs with a lipid concentration of 55% and higher; with 600 rpm, for 2 min, and at 20 °C. In the third step, the calcein/VPG mixtures were diluted with TRIS buffer by DC (500 rpm, 2 min, and 20 °C). The amount of calcein, that was taken up into the liposome formed from the open membrane stacks structures, was determined by encapsulation efficiency (EE) measurements by time-resolved fluorescence as described in a previous study [2]. Data are shown as the mean ± SD (n = 3).

After the addition of 60 mM calcein buffer to the empty VPGs made without calcein, EE was determined by TRF (Figure 4). For HEPC/Chol 55/45, the clearly identifiable uptake of calcein into liposomes formed from open membrane stack structures started at VPGs prepared at 70% lipid concentration. For HEPC/Chol/DSPE-PEG2000 50/45/5, the uptake started at lower lipid concentrations of about 50%, as expected due to the water-binding properties of the PEG chains. Furthermore, the uptake increased over a broader lipid concentration area.

#### 3.1.5. Liposome Formation without Lipid Film Preparation in Organic Solvents

**Figure 5:**
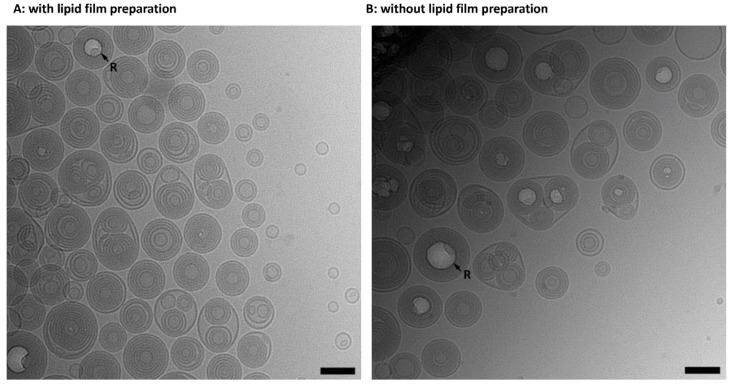
HEPC/Chol 55/45 (mol%) liposomes prepared with (**A**) and without (**B**) lipid film preparation in organic solvents. Preparation at 60% lipid concentration by DC at 2350 rpm, at 20 °C, and for 30 min. Arrows (R) indicate radiation damage due to the dense material nature of the multilamellar liposomes. Scale bar: 100 nm.

So far, only DSC measurements were used to show that VPGs/liposomes can be prepared from lipid mixtures by DC-homogenization without preparing a lipid film from an organic lipid dissolution in advance [2]. Thus, here we compared liposome formation using cryo-EM images of liposomes (60% lipid concentration, HEPC/Chol 55/45) prepared by DC with and without prior lipid film preparation (Figure 5). Cryo-EM images of liposomes prepared with (Figure 5A) and without prior lipid film preparation (Figure 5B) showed the same lamellarity and the same liposome sizes. No cholesterol crystals were visible in either sample. To the best of our knowledge, we showed for the first time, by cryo-EM imaging, that liposomes can be prepared by DC without the use of any organic solvents.

### 3.2. Morphology of Cholesterol-Free Liposomes

#### 3.2.1. Investigations Based on IAS Measurements

**Figure 6:**
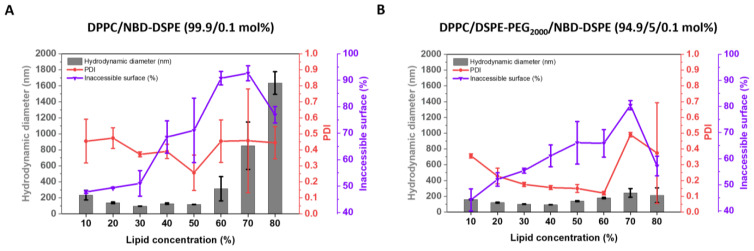
IAS-determination of cholesterol-free liposomes. (**A**,**B**): IAS-assay and DLS results of DPPC/ NBD-DSPE 99.9/0.1 (mol%) (**A**) and DPPC/DSPE-PEG2000/NBD-DSPE 94.9/5/0.1 (mol%) (**B**) of DC prepared liposomes (2350 rpm, 40 °C, and 30 min) dependent on the lipid concentration (10–80%) used for the DC-homogenization of the lipid/buffer mixture. The resulting VPGs were redispersed with TRIS buffer at 1500 rpm, for 2 min, and at 20 °C. The liposomes were prepared and diluted in TRIS buffer containing 25 mM NaCl. To quench the accessible surface, sodium dithionite was added (10 mM) to liposomes diluted in a buffer (0.05 mM lipid). Data are shown as the mean ± SD (n = 3).

The IAS for liposomes made from pure DPPC increased steadily in the interval from 10% to 70% lipid concentration, reaching a maximum of about 90% IAS at 70% lipid concentration (Figure 6A). In contrast to HEPC/Chol (Figure 2A), the IAS then decreased for samples with 80% lipid concentration. This is probably due to a shortage of water during the DC-homogenization since it requires more water to hydrate DPPC in contrast to the same mass of HEPC/Chol. For lipid concentrations <30% and >50%, the DPPC liposomes tend to be larger in size and more polydisperse compared to the cholesterol-containing vesicles. At 80% lipid concentration, the DPPC liposomes form large structures (>1 µm), which again can be explained by a shortage of water during DC-homogenization.

DPPC liposomes in the presence of 5 mol% PEG (Figure 6B) also showed an increasing IAS with increasing lipid concentration from 10% to 70%. As already seen for the incorporation of PEG into HEPC/Chol liposomes (Figure 3A), the incorporation of PEG-lipids led to a lower IAS (Figure 6B). From 70% to 80% lipid concentration, the IAS decreased substantially from about 80% to about 55%. An increase in size for lipid concentrations <20% and >60% was detected but less pronounced than for the PEG-free DPPC liposomes. 

#### 3.2.2. Investigations Based on Cryo-EM Images

**Figure 7:**
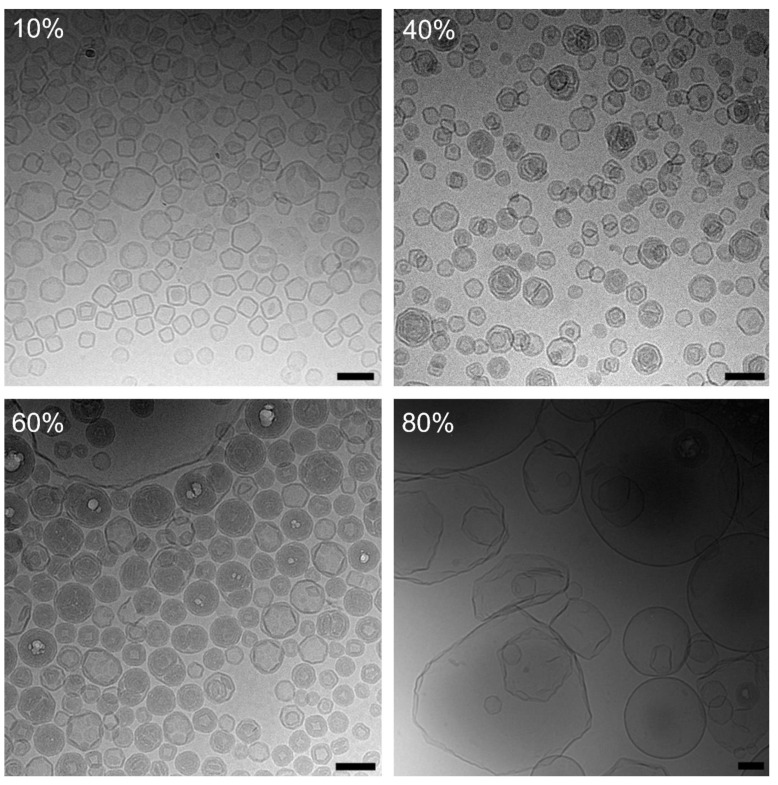
Cryo-EM images of DPPC-liposomes prepared by DC at 10%, 40%, 60%, and 80% lipid concentrations (VPG) at 2350 rpm, at 20 °C, and for 30 min with TRIS buffer and redispersed with the same buffer by DC at 1500 rpm, at 20 °C, and for 2 min. In-vial temperature after preparation was about 40 °C. Scale bar: 100 nm.

The cryo-EM images shown in Figure 7 display the structures found in liposomal dispersions originating from DPPC-based VPGs produced at increasing lipid concentrations. As observed for the cholesterol-containing dispersions, samples prepared using 10% lipid concentrations were clearly dominated by unilamellar liposomes. Notably, the liposomes were not perfectly spherical but displayed the angular shape typically exhibited by PC-liposomes at temperatures below T_m_ [19,20,21]. Images captured from samples prepared with 40% and 60% lipid concentrations revealed an increasing number of multilamellar liposomes with varying sizes up to about 200 nm. In the case of samples prepared at 60% lipid concentration, the analyses disclosed the presence of some large unilamellar liposomes with diameters around 500 nm. Samples prepared with 80% lipid concentration displayed no multilamellar liposomes but were instead dominated by large unilamellar liposomes.

## 4. Discussion

### 4.1. Fluorescence-Based Lamellarity Assay—Opportunities and Limitations

The method used here to characterize the lamellarity of liposomal vesicles was adapted from Gruber et al. [16] and is based on the incorporation of a lipid-bound fluorophore (NBD-DSPE) on the membrane surfaces during vesicle preparation. Subsequent quenching of the fluorophore by dithionite occurs only at the accessible, outer surfaces [16], while the inaccessible NBD-fluorophores inside the vesicles stay untouched. The inaccessible fraction of the surfaces can be exactly calculated but the lamellarity—the exact number of lamellae of a vesicle—can only be “estimated” from the inaccessible surfaces. In NMR measurements to determine lamellarity [22], which work in a comparable way by shifting the ^31^P-Signal of the phospholipid headgroups at the outer (accessible) surfaces of liposomal membranes by shift-reagents such as Pr^3+^ or Eu^3+^, lamellarity (L) was simply calculated by the equation: L = 100/(2 × relative loss of signal) or by L = (peak area of both peaks)/(peak area of shifted peak) [22]. However, having rather small liposomes and a typical membrane thickness of about 3–4 nm, lamellarity-values are systematically underestimated using this formula, even with unilamellar vesicles, since the inner surface area is significantly smaller than the outer surface area. Unfortunately, the problem of underestimation becomes even more crucial for multilamellar vesicles (which we are particularly interested in in this study) since the surface areas of the inner “liposomes” become smaller the more membranes a vesicle has. Taken together, it is per se not possible to accurately calculate lamellarity from the inaccessible surface data. Instead, in this study, IAS was used to describe the morphology of the vesicles.

Since it was found that IAS cannot only be determined using the very sensitive, single photon counting FT100 but also with normal steady-state fluorometers such as MTP- or cuvette-fluorescence readers using the identical liposomal dispersion, the assay supplements the already described liposomal formulation screening process [2] in a very easy way. This advantage compensates for the drawback that the fluorescently labeled lipids must be added to the lipid blend at the beginning of the whole screening process and that the label—despite its very low concentration—might marginally influence the liposomal characteristics.

### 4.2. Tailoring the Lamellarity of Small Liposomes

The DC-homogenization of moderately concentrated lipid/buffer mixtures (typically up to 20% or 30% lipid concentration) resulted in rather small, as well as unilamellar vesicles, as shown by the lamellarity assay and cryo-EM. Thus, it can be concluded that the formation of unilamellar vesicles within a VPG is preferred over multilamellar vesicles, probably driven partly by the high energy demand of forcing the rather rigid HEPC/Chol-membranes into structures with high curvature—as it is needed for the formation of small multilamellar vesicles. If excess water is available, unilamellar liposomes would be preferred over multilamellar liposomes for entropic reasons as well.

Reaching about 30% lipid concentration, the steep increase in the EE-values transformed into a flatter increase [2], which can be explained by the assumption that at this point, the densest packing of unilamellar vesicles is reached and that the densely packed vesicles already enclose most of the water phase containing the hydrophilic marker or drug. A rough calculation based on unilamellar vesicles of the same 140 nm size reveals that at 20% lipid concentration, the maximum tight packing of vesicles is reached within the primarily formed VPG, which is in slight contrast to our findings. However, this discrepancy can be explained by the heterogenicity of the vesicles (PDI 0.2–0.3) at those lipid concentrations, with a tendency towards a bigger portion of smaller vesicles as shown by cryo-EM (compare Figure 2 and Figure 3).

At lipid concentrations higher than about 30%, there was more lipid available than could be accommodated as unilamellar vesicles in a certain volume. In an earlier study [2], it was discussed that the resulting vesicles within the primarily formed VPGs might no longer be round but rather lentil- or erythrocyte-like shaped, which allows for denser packing compared to ball-like vesicles. Kaiser et al. [23] showed cryo-EM pictures of very small and some lentil-like vesicles after using a comparable 30%-lipid mixture for making VPGs/liposomes by using high-pressure homogenization. In contrast to that, in this study, the resulting liposomes are perfectly spherical after dilution of the respective VPGs (as shown by cryo-EM, compare Figure 2 and Figure 3). If lentil- or erythrocyte-like lipid vesicles had existed within the VPGs, water must have diffused into and inflated these deformed vesicles upon dilution. However, that would be in contradiction to our finding that the concentration of the entrapped calcein within the redispersed liposomes was the same as the calcein concentration used for the initial VPG preparation, showing that no dilution took place due to the diffusion of water into the vesicles. The calcein-concentrations within the liposomes have been measured by their fluorescence lifetimes (τ-values, published in [2]).

Increasing the lipid content during VPG production to 30% and higher resulted in liposomes of similar hydrodynamic size but with more lamellae. Thus, since the vesicles within the VPGs produced at concentrations higher than about 30% were round and had sizes similar to the vesicles that resulted from lower lipid concentrations, we concluded that the excess of lipid at higher lipid concentrations was probably located inside the vesicles, resulting in small multilamellar vesicles. This conclusion was supported by cryo-EM pictures, as well as by the lamellarity assay which showed an increasing inaccessible surface (IAS) with increasing lipid concentrations. Our findings agree with the findings of Tardi et al. who showed freeze-fracture electron microscope pictures of vesicles prepared by high-pressure homogenization that clearly display the expected multilamellar vesicular structures [24].

As shown by the IAS-assay and cryo-EM pictures, at a concentration of about 60%, the maximal lamellarity was reached, meaning that the vesicles were completely filled with membranes (Figure 2B, 60%) which are all in close vicinity. The EE-values for water-soluble compounds decreased somewhat (from 70% to 60% [2]), which could be explained by the reduced aqueous compartment between the lamellae inside the SMVs.

Liposomes are typically classified by the number and arrangement of bilayers entrapping an aqueous core. For multilamellar liposomes, a distinction between multilamellar (MLVs) and multivesicular vesicles (MVVs) was made. For unilamellar vesicles, three classifications according to their size are common: giant unilamellar vesicles (GUVs; >1 µm), large unilamellar vesicles (LUVs; 200 nm to 1 µm), and small unilamellar vesicles (SUVs; 20 to 200 nm) [25]. MLVs and MVVs are typically in a size range from 500 nm to 5 µM [25]. Small multilamellar vesicles (SMVs) in a size range from about 100 to 500 nm have not been classified so far, since it has not been possible to intentionally and reproducibly produce multilamellar vesicles of a small size. Instead, the formation of multilamellar vesicles is typically an unwanted result during the preparation of unilamellar vesicles. Regardless of this, small multilamellar vesicles (SMVs) are of high interest as drug carriers for lipophilic or amphiphilic drugs, and as slow-release drug carriers for hydrophilic drugs, including proteins, peptides, and nucleic acids. Our results clearly show that unilamellar-vesicles and SMVs of about the same size can easily be produced by simply adjusting the lipid concentration during VPG preparation by DC. The high EE-values found for the entrapment of calcein into unilamellar vesicles, as well as SMVs [2], show that both types of liposomes can be used to efficiently entrap water-soluble drugs including siRNA [26,27]. Using SMVs has the potential advantage of an expectedly slower release of drugs compared to unilamellar vesicles since more membranes must be crossed or removed. In addition, the small size of the SMVs potentially enables them to accumulate in tumor tissues due to the EPR-effect [28,29].

Due to their higher membrane content, SMVs are also promising carriers for lipophilic or amphiphilic drugs. The higher vesicle stiffness caused by the additional lamellae of SMVs seems to improve their cellular uptake [30]. This increase in stiffness due to multilamellarity might also contribute to the impressively low PDI-values systematically found for SMVs. During DC-homogenization, the collision of stiffer or less flexible particles might result in the removal of outer membranes until the remaining core is too stiff to become further homogenized, which might also be supported by the cooperative behavior of the very closely spaced lamellae of the SMVs. Cryo-EM pictures of those formulations show either SMVs full of membranes or, to a small extent, unilamellar-vesicles, but nothing in between, which supports the above-made assumption.

The expected slow release of water-soluble drug compounds makes SMVs also promising drug carriers within depot formulations, for intramuscular, intraperitoneal, or subcutaneous applications. Due to the fact that those depot formulations have to be injected by syringes using thin needles, the currently used VPG-based depot-formulations are made at rather low lipid concentrations [1,31]. This has the advantage of low viscosity, but the vesicles are expected to be rather unilamellar—a diluted formulation of SMVs appears as a promising alternative.

Based on the idea that it is mainly the lipid concentration used for VPG preparation that determines the formation of unilamellar vesicles or SMVs, it is conceivable that other techniques such as HPH which are basically suitable to prepare VPGs will result in comparable vesicles after dilution of the VPGs. One disadvantage of HPH in contrast to DC is that the resulting VPGs usually have to be autoclaved prior to use which results in an unwanted increase in the size of the final vesicles [24]. Due to that and because the lipid concentration range that can be processed by HPH is typically limited to about 40% to 50% lipid concentration [24,32], the use of HPH to prepare SMV via VPGs needs to be investigated in a separate study.

### 4.3. The Shortage of Water Limits the Formation of Closed Vesicles

At very high lipid concentrations such as >65% for HEPC/Chol-mixtures, DC-homogenization results in large and heterogenous vesicles with decreasing EE-values. Let us estimate the minimal amount of water needed to maintain closed multilamellar structures: Approximately 12 water molecules are necessary to hydrate a phospholipid headgroup [33,34,35] which corresponds to a VPG consisting of 78% pure phospholipid and 22% water. In such a VPG all water is bound to the headgroups and localized between the headgroups and in the tiny water layer between the “tightly” stacked lamellae.

Closed vesicular structures, however, need additional water to fill the interstices between the vesicular structures and their inner core. For densely packed, equally sized spheres, the theoretical fraction of interstitial space is 26 v%. This fraction is reduced for polydisperse, deformable bodies but for interstices plus cores, we may still estimate a space requirement of 10–30 v%. Adding this to the 22% PC-bound water (s. above) results in a minimal amount of water of about 32–52% (or a maximum lipid concentration of 48–68%) which is necessary to form a DPPC-VPG consisting of closed vesicular structures. Since the vesicles within a typical VPG are typically heterogeneous in size and lamellarity, one must expect the appearance of open stacks of lamellae coexisting of closed structures in VPGs of 48–68% lipid concentration for pure DPPC-VPGs. Irrespective of the heterogeneity of the vesicles, at about 70% phospholipid, no closed structures are expected to exist anymore.

However, the HEPC/Chol-mixture used in this study to produce VPGs consisted of only 71.4% phospholipid and 28.6% cholesterol, which binds less water than phospholipids, meaning (i) that the formation of open lipid stacks will start at somewhat higher lipid concentrations and (ii) that closed vesicles are also expected at somewhat higher lipid concentrations. The experimental finding that the amount of open lipid stacks is maximized at 78% lipid concentration for HEPC/Chol-VPGs is therefore in good agreement with the above-made hypothesis.

Diluting calcein-free VPGs with a calcein solution resulted in an increased entrapment of the added dye (Figure 4), starting at about 65% lipid (10% entrapment) and reaching 80% entrapment at 80% lipid. This corresponds to an increasing fraction of open lamellar stacks in the original VPG that re-close and thus entrap calcein solution upon dilution. Vice versa, since the calcein-containing VPGs were usually diluted with non-calcein-containing buffer, the EE-values at concentrations >65% should decrease, an effect that could clearly be observed in a previous study [2].

However, the low uptake of calcein noted at lower lipid concentrations can be interpreted as an artifact of the EE-calculation routine [2]. Specifically, when only low concentrations of calcein are entrapped, the biexponential fitting of the lifetime decays may fail to precisely distinguish between entrapped and non-entrapped calcein.

Cryo-EM pictures of VGPs made from 80% HEPC/Chol confirmed the existence of large multilamellar vesicles but also showed numerous vesicle-in-vesicle structures. The fact that no open membrane stacks and only closed structures are visible in the cryo-EM-pictures can be explained by the dilution of the highly concentrated VPGs that was necessary to prepare the samples for cryo-EM. The re-closed open stack structures are the result of a lack of water during the DC-homogenization process. Following this assumption, no homogenous liposomes can be formed anymore, and only large non-homogeneous vesicles are formed. This can be observed by the sharp increase in the size and the size distribution (increasing PDI) of the vesicles prepared by DC-homogenization between 70% and 80% lipid concentration (Figure 2A).

In accordance with the fact that PEG chains bind additional water molecules, the uptake of calcein in open lipid structures started at somewhat lower lipid concentrations (about 50%) when using a lipid-mixture containing 5 mol% DSPE-PEG_2000_. The curvature-active PEG lipid may also stabilize the dehydration-induced open lamellar stacks by covering their open edges—at a low PEG-lipid content, they would redistribute over the membrane as the sheets re-close upon dilution.

In the case of HEPC/Chol liposome dispersions prepared by conventional methods, it has previously been shown by cryo-EM that the inclusion of 5 mol% PEG-lipid induces the formation of a small population of lipodisks [36]. The lipodisk-to-liposome ratio increases with increasing PEG-lipid concentration, and at a PEG-lipid content of 20 mol%, the samples are typically completely dominated by lipodisks [18,36,37,38]. Cryo-EM pictures showed the presence of a quite significant population of lipodisks in the PEG-containing samples prepared by DC (Figure 3B). The formation of disks, which are two-dimensional structures that do not entrap water-soluble compounds, might explain why the EE-values are significantly lower when using lipid mixtures containing PEG—in addition to the fact that the unstirred water layer on the membranes also reduces the inner volume of the vesicles. Furthermore, the existence of lipodisks might also contribute to the much lower lamellarity-values found for PEG-containing lipid mixtures at higher lipid concentrations, while at lower lipid concentrations, the EE-values are comparable. Since the IAS assay is based on quenching the fluorescence signal at all accessible surfaces, the amount of accessible surfaces is much higher when lipodisks are present. A perhaps even more important reason for the lower lamellarity values found in the presence of PEG-lipid is that the steric stabilization conferred by the PEG polymers effectively hinders the dense packing of the lipid lamellae inside the MLVs. The difference in spacing is clearly seen by a comparison of the cryo-EM micrographs displayed in Figure 2B and Figure 3B.

## 5. Conclusions

The newly developed in-vial homogenization using dual centrifugation (DC) is by far the easiest and fastest way to prepare liposomes from various lipid compositions, but it is not fully understood in detail. Here, we identified two novel liposomal preparations with considerable potential for pharmaceutical application. Further, in addition to largely unilamellar vesicles, we were able to produce small multilamellar vesicles (SMVs) as well as self-entrapping open bilayer stacks that can be stored and then used to spontaneously entrap an aqueous solution of a drug that is being added before administration.

**Figure 8:**
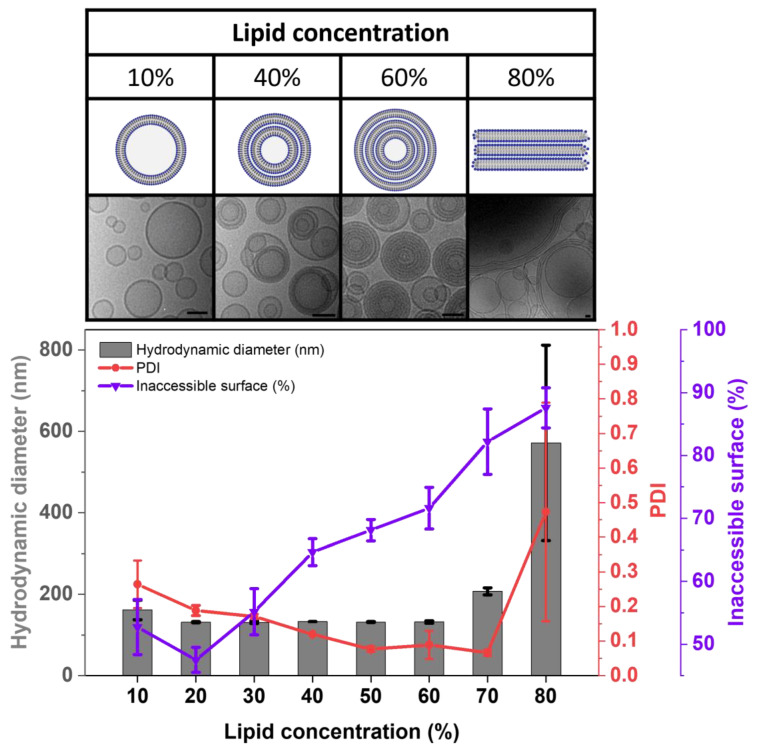
Summary of the observed changes in morphology, size, size distribution, and the inaccessible surface upon increasing the lipid concentration used for VPG preparation by DC. Cartoons (top of figure) illustrate the postulated lipid structures present in the VPGs before dilution. Cryo-EM images correspond to samples composed of HEPC/Chol 55/45, whereas samples composed of HEPC/Chol/NBD-DSPE 54.9/45/0.1 were used in the IAS-assay and DLS measurements. Liposomes were prepared by DC at 2350 rpm, for 30 min, and at 20 °C with TRIS buffer, and the resulting VPG was diluted with TRIS buffer to a liposomal dispersion at 1500 rpm, for 2 min, and at 20 °C. DLS and LL data are shown as the mean ± SD (n = 3). Scale bar = 50 nm in cryo-EM pictures.

In more detail, the investigation of the lamellarity of DC-made liposomes using cryo-EM and a fluorescence-based lamellarity assay substantially improved our understanding of the vesicle-forming processes and the morphology of the resulting liposomes. The insights gained concerning the structural behavior of DC homogenized lipid mixtures composed of HEPC/Chol 55/45 are summarized in Figure 8. At low lipid concentrations of 10%, the viscosity of the lipid/water mixture is low during homogenization, resulting in rather weak shear forces and, thus, slightly larger, and more heterogenous vesicles. From about 20% lipid concentration on, the liposomes become smaller and more homogenous. As shown by cryo-EM, the formation of unilamellar vesicles is preferred over multilamellar vesicles, at least for liposomes made from lipid mixtures that result in rather rigid membranes.

Further increasing the lipid concentration to 40%—which is more lipid than can be accommodated in densely packed, unilamellar liposomes—had only a minor influence on the already small vesicle size, however, multilamellar and multivesicular vesicles then became dominant. At about 60% lipid concentration, the lamellarity was at its maximum. Cryo-EM pictures show vesicles almost completely filled with membranes that are very closely packed. The very narrow size distributions are probably due to the higher stability of the multilamellar vesicles against shear stress during homogenization.

At about 65% lipid, there is not enough water to fully hydrate the phospholipid headgroups and fill interstices between and the remaining cores within vesicles. With increasing lipid content in the VPG, closed vesicular structures are increasingly replaced by open lamellar stacks that re-close and, in this way, entrap the calcein solution added upon dilution. These re-formed vesicles are rather big and heterogeneous. At about 80% lipid, the remaining 20% water is almost completely needed as lipid-bound, interlamellar water, and virtually no closed structures are left in the gel.

Adding PEG-containing lipids to the lipid mixture caused the formation of open membrane sheets already at medium lipid concentrations, an effect which was likely due to the high water-binding capacity of PEG and the stabilization of the edges of the open membrane sheets by PEG.

In addition to providing a better understanding of the processes during liposome preparation using DC, the new findings allow for the specific tailoring of unilamellar as well as small multilamellar vesicles (SMVs) with encouraging entrapping efficiencies for water-soluble as well as lipophilic compounds by simply adjusting the lipid concentration during DC.

## Data Availability

Not applicable.
